# The impact of antimicrobial stewardship program implementation at four tertiary private hospitals: results of a five-years pre-post analysis

**DOI:** 10.1186/s13756-020-00751-4

**Published:** 2020-06-29

**Authors:** Awad Al-Omari, Abbas Al Mutair, Saad Alhumaid, Samer Salih, Ahmed Alanazi, Hesham Albarsan, Maha Abourayan, Maha Al Subaie

**Affiliations:** 1 Research Center, Dr Sulaiman Al Habib Medical Group, Riyadh, Saudi Arabia; 2 Alfaisal University, Riyadh, Saudi Arabia; 3 School of Nursing, Wollongong University, Wollongong, Australia; 4 Ministry of Health, Canberra, Australia

**Keywords:** Antimicrobial agents, Broad-spectrum antibiotics, Consumption, Cost, Defined daily dose, Healthcare associated infections, Saudi Arabia

## Abstract

**Background:**

Antimicrobial stewardship (AMS) programs have shown to reduce the emergence of antimicrobial resistance (AMR) and health-care-associated infections (HAIs), and save health-care costs associated with an inappropriate antimicrobial use. The primary objective of this study was to compare the consumption and cost of antimicrobial agents using defined daily dose (DDD) and direct cost of antibiotics before and after the AMS program implementation. Secondary objective was to determine the rate of HAIs [*Clostridium difficile* (*C. difficile*), ventilator-associated pneumonia (VAP), and central line-associated bloodstream infection (CLABSI) before and after the AMS program implementation.

**Methods:**

This is a pre-post quasi-experimental study. Adult inpatients were enrolled in a prospective fashion under the active AMS arm and compared with historical inpatients who were admitted to the same units before the AMS implementation. Study was conducted at four tertiary private hospitals located in two cities in Saudi Arabia. Adult inpatients were enrolled under the pre- AMS arm and post- AMS arm if they were on any of the ten selected restricted broad-spectrum antibiotics (imipenem/cilastatin, piperacillin/tazobactam, colistin, tigecycline, cefepime, meropenem, ciprofloxacin, moxifloxacin, teicoplanin and linezolid).

**Results:**

A total of 409,403 subjects were recruited, 79,369 in the pre- AMS control and 330,034 in the post- AMS arm. Average DDDs consumption of all targeted broad-spectrum antimicrobials from January 2016 to June 2019 post- AMS launch was lower than the DDDs use of these agents pre- AMS (233 vs 320 DDDs per 1000 patient-days, *p* = 0.689). Antimicrobial expenditures decreased by 28.45% in the first year of the program and remained relatively stable in subsequent years, with overall cumulative cost savings estimated at S.R. 6,286,929 and negligible expenses of S.R. 505,115 (*p* = 0.648). Rates of healthcare associated infections involving *C. difficile*, VAP, and CLABSI all decreased significantly after AMS implementation (incidence of HAIs in 2015 compared to 2019: for *C. difficile*, 94 vs 13, *p* = 0.024; for VAP, 24 vs 6, *p* = 0.001; for CLABSI, 17 vs 1, *p* = 0.000; respectively).

**Conclusion:**

Implementation of AMS program at HMG healthcare facilities resulted in reduced antimicrobials use and cost, and lowered incidence of healthcare associated infections.

## Introduction

For decades microbes, in particular bacteria, have become increasingly resistant to various antimicrobials. The World Health Assembly’s endorsement of the Global Action Plan on Antimicrobial Resistance (AMR) [1] in May 2015, and the Political Declaration of the High-Level Meeting of the General Assembly on AMR [2] in September 2017, both recognize AMR as a global threat to public health. These policy initiatives acknowledge overuse and misuse of antimicrobials as a main driver for development of resistance, as well as a need to optimize the use of antimicrobials.

Antimicrobial stewardship (AMS) is a coherent set of actions which promote the responsible use of antimicrobials. This definition can be applied to actions at the individual level as well as the national and global level, and across human health, animal health and the environment [3, 4]. Antimicrobial stewardship programs optimize the use of antimicrobials, improve patient outcomes, reduce AMR and health-care-associated infections (HAIs), and save health-care costs amongst others [5, 6].

Many countries around the world have developed and are implementing their national action plans (NAPs) on AMR, [7] in which AMS is a key priority. Although there is a scientific evidence base for AMS, [8] and national, regional and global guidance documents exist, [9–13] there is a growing need for more specific guidance on how to establish, implement and evaluate effective AMS programs at the national and health-care-facility level [11, 14].

AMS programs result in significant decreases in antimicrobial consumption and cost and improve infections due to specific antimicrobial-resistant pathogens and the overall hospital length of stay as well [5, 6]. Future studies should focus on the sustainability of these outcomes and evaluate potential beneficial long-term effects of AMS programs in mortality and infection rates [15].

As stewardship programs can lower unnecessary consumption and cost of antimicrobials [5, 6], Al Habib Medical Group (HMG) established, created and implemented a stepwise AMS program and guidelines for antimicrobial use within its healthcare facilities.

## Study purpose

This study aimed to measure AMS program impact by comparing a One-year baseline period prior to the implementation of the AMS to Four years of follow-up data after the program was initiated in terms of restricted antimicrobials use and cost, and rate of healthcare associated infections (HAIs) occurrence in adult inpatients hospitalized at four HMG healthcare facilities (Olaya, Altakhassusi, Arryan and Qassim) in Saudi Arabia.

## Settings

Habib Medical Group (HMG) is globally distinguished as one of the largest healthcare providers of comprehensive healthcare services in the Middle Eastern region and aims towards providing excellence in various specialized healthcare services to fulfill all patients’ needs. Therefore, the medical group is currently operating 22 medical facilities across Saudi Arabia, United Arab Emirates UAE and Bahrain, including 7 hospitals and 6 medical centers. All HMG medical facilities are working according to the highest international standards.

Study was conducted at four HMG tertiary and specialized health facilities with adequate medical professional resources including: Dr. Sulaiman Al Habib Olaya Medical Complex: a 195-bed capacity, Dr. Sulaiman Al Habib Hospital in Altakhassusi: a 237-bed capacity, and Dr. Sulaiman Al Habib Arryan Hospital: a 365-bed capacity, in Riyadh; and Dr. Sulaiman Al Habib Qassim Hospital: a 150-bed capacity, in Qassim, Saudi Arabia.

These facilities provide healthcare to a wide range of patients in various specialties and subspecialties, including maternity and children, general medicine and surgery, cardiac surgeries, bone, joint and spine surgery, dermatology and plastic surgery, ophthalmology and laser or vision correction surgery, neurosurgery, obesity surgery, intensive care unit, dialysis, hematological and solid organ malignancies, and sports injuries. Yearly, these four healthcare facilities encounter over 152,995 surgical cases, nearly 2,152,441 emergency department visits, and over 409,403 admissions.

## AMS program

AMS program has been implemented at HMG medical settings since January 2016 to optimize the use of antibiotics; reduce further emergence, selection and spread of AMR; decrease the consumption of broad-spectrum antimicrobials; reduce the rate of HAIs and other multidrug resistant organisms; and save on unnecessary health-care costs.

HMG medical facilities adapt different types of AMS interventions to improve antibiotic prescribing have proven successful [5]. These AMS interventions include education (prescribers and other healthcare workers) [16], and/or audit and feedback activities (real-time, either written or oral, or retrospective), [17] and/or restrictive interventions, such as pre-authorization of targeted antibiotics [18]. Restrictive interventions, a core strategy that provide the foundation for an AMS program, have been shown to provide immediate and significant reduction in antibiotic use and cost [17, 18]. All four HMG facilities restrict a group of antibiotics that should be reserved for treatment of confirmed or suspected infections due to multi drug-resistant organisms, and treated as “last-resort” options. Use of restricted antibiotics may be limited to certain indications, prescribers, services, patient populations or a combination of these. A practical approach that allows attending physician to use the drug pending approval by physician and/or pharmacist or AMS team after +/− 48 h.

## Methods

### Study design

A pre-post quasi-experimental study design was used to analyze the clinical outcomes of the AMS by comparing antimicrobial utilization and cost, and rate of HAIs occurrence data for designated periods before (January 2015– December 2015) and after (January 2016–June 2019) AMS program initiation in adult inpatients hospitalized at four HMG medical facilities in Saudi Arabia: Olaya, Altakhassusi, Arryan and Qassim.

### Data collection

In January 2015, we began using data-mining software to develop automated reports to capture key pharmacy, cost and microbiological data on all adult hospital inpatients receiving those the following 10 restricted antimicrobials: imipenem/cilastatin, piperacillin/tazobactam, colistin, tigecycline, cefepime, meropenem, ciprofloxacin, moxifloxacin, teicoplanin and linezolid. Data were collected to evaluate the impact of AMS interventions (baseline and follow-up data) on use of those 10 restricted antibiotics to identify areas for improvement of antibiotic prescribing for the whole HMG organization, a department or ward; and to provide regular and structured feedback both on the quality and quantity of antibiotic prescribing and use to prescribers. Data of a five-year analysis were compared for time-related changes in antimicrobials consumption and cost, and HAIs incidence rate pre- and post- AMS program implementation.

Data were collected in separate customized Excel spreadsheets (version 2019; Microsoft Corp, Redmond, WA). Antibacterials that were high cost, high use, or required preservation due to the potential for resistance issues to develop were included. No data for restricted antimicrobials use in adult inpatients in all departments and units at the targeted four HMG health facilities were excluded. All adult patients admitted during the study period and received at least one dose of one of the restricted antimicrobials were included. Exclusion criteria included age less than 18 years, consumption of antimicrobials not included in the study, and consumption of antimicrobial by a route other than parenteral or oral routes.

Data regarding drug consumption were gathered from our electronic prescribing records, pharmacy dispensing data and electronic medication administration records and reported in terms of defined daily doses (DDDs), as recommended by the WHO Collaborating Centre for Drug Statistics Methodology [19]. Antimicrobial utilization was calculated based on the defined daily dose per 1000 acute patient days (DDD/1000 patient days). The DDD per 1000 patient days was calculated by dividing the cumulative use of a specific antimicrobial (in grams) by a defined daily standard dose for that drug and then expressing it per every 1000 acute patient days.

The acquisition costs of all parenteral and oral antimicrobial agents given to adult inpatients were collected and compared over a five-year time frame. Current acquisition prices were used for all cost comparisons to account for any changes in those costs over the studied period. Antimicrobial cost data included purchases made through the hospital’s pharmaceutical wholesaler and direct purchases from the manufacturer. Total expenditures for all antimicrobials were calculated and divided by the applicable total number of patient-days to derive a figure for “Antimicrobial Riyals Per Patient Day” (ARPD). Actual cost savings related to the AMS were calculated by subtracting the ARPD for each of the four study years (2016–2019) from the ARPD for 2015, the baseline year (i.e., the year before the start of the AMS); each difference was then multiplied by the number of patient-days for the specific year.

The impact of the AMS program on HAIs involving *Clostridium difficile* (*C. difficile*), ventilator-associated pneumonia (VAP), and central line-associated bloodstream infection (CLABSI) was determined by following the occurrence rates before and after AMS program launch. Sources of gathered data on HAIs incidence were retrieved from the microbiology, epidemiology, and infection control surveillance databases.

The institutional review board at HMG approved this study as exempt from the full board review and waived the need for informed consent because of the use of existing data and the information being recorded in a way that subjects cannot be individually identified.

### Statistical analysis

Continuous variables were presented as sums and means. Categorical variables were presented as frequencies and percentages. Descriptive statistics, testing for changes in antibiotic use and cost and rate of HAIs incidence data was made by simple calculations, estimating frequency and percentage differences between two or more values, and creating combo charts were made by Excel (version 2019; Microsoft Corp, Redmond, WA). The pre- and post-AMS periods were compared for time-related changes in HAIs, antimicrobial use, and ARPD by analyzing analysis of variance (ANOVA). The overall levels of infection were compared between the pre- and post-AMS periods through analysis of variance. The a priori level of significance was 0.05.

## Results

There was little variation during the study period with respect to the total number of hospital admissions in all HMG four facilities (79,369 in 2015, 80,586 in 2016, 78,257 in 2017, 82,049 in 2018, and 89,142 in 2019), and emergency room visits (364,167, 456,224, 399,139, 433,616 and 499,295), reflecting a relatively stable inpatient hospital population during the time period evaluated.

A total of 409,403 patients were involved in our study, among whom 79,369 patients were involved before the AMS program implementation corresponding to 163,024 patient days and an average of 330,034 patients were involved after the AMS interventions making up an average of 7,788,385 patient days between 2016 to 2019.

**Fig. 1:**
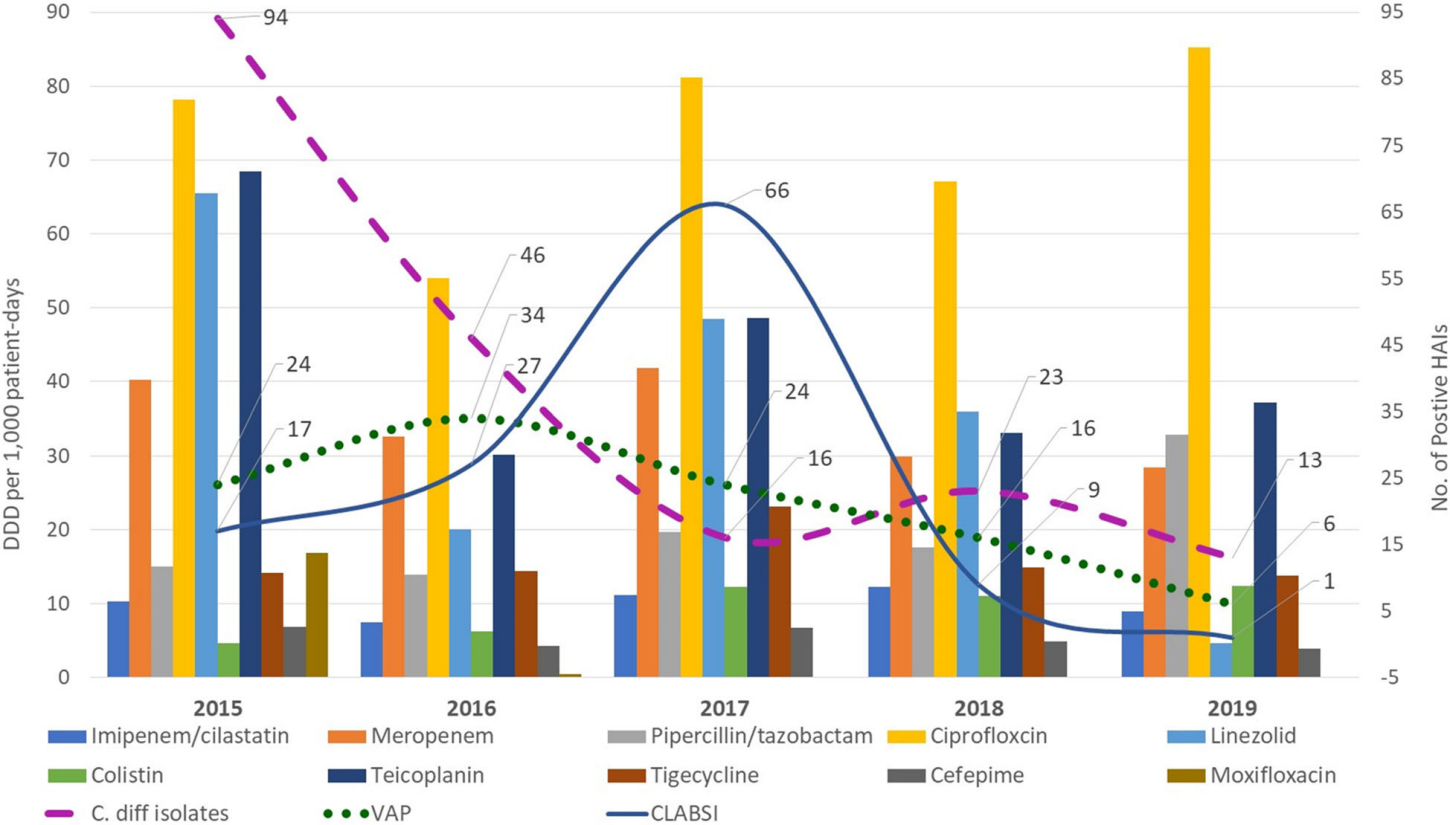
Restricted antimicrobials use and rate of HAIs occurrence before and after implementation of AMS program in all four HMG medical facilities, 2015–2019. Abbreviations: c. diff, *Clostridium difficile*; CLABSI, central line-associated bloodstream infection; DDD, defined daily dose; HAIs, healthcare associated infections; VAP, ventilator-associated pneumonia

Antimicrobial consumption, expressed as DDDs per 1000 patient-days, had risen in the years before the AMS program launch. Individual restricted antimicrobial consumption before and after AMS program beginning and rate of HAIs incidence is shown in Fig. 1. The most consumed antimicrobial agents (as total of DDDs in all facilities over 5 years) included ciprofloxacin (365 DDDs), teicoplanin (217 DDDs), linezolid (175 DDDs), and meropenem (173 DDDs). The least frequently consumed antimicrobial agents (as total of DDDs in all facilities over 5 years) included moxifloxacin (17 DDDs), cefepime (27 DDDs), colistin (46 DDDs), and imipenem/cilastatin (50 DDDs). Almost all (100%) antimicrobial consumption was administered parenterally.

As shown in Fig. 1, the use of almost all antibiotic classes trended downward in the year after AMS program commencement; the year to year change in DDD values did not differed significantly between the pre- and post- AMS periods (*p* = 0.689).

Notably, average total linezolid use in the one pre-AMS year was 66 DDDs per 1000 patient-days; overall linezolid use fell by to an average of 70% to an average of 20 DDDs per 1000 patient-days. Similarly, teicoplanin use reduced from 68 to 30 DDDs per 1000 patient-days (a 56% decrease of drug’s average total consumption). Ciprofloxacin was the most used antibiotic of all selected agents and its use steadily rose to a peak of 85 DDDs per 1000 patient-days in 2019. Imipenem/cilastatin, tigecycline and cefepime were almost consumed at equal DDDs throughout the study period. Moxifloxacin consumption declined from 17 DDDs per 1000 patient-days to zero post AMS implementation and remained at this nil level on the following years.

Before the AMS intervention, we observed a high incidence of *C. difficile*, VAP, and CLABSI, which were the most common of HAIs in the targeted HMG medical facilities. HAIs rates for *C. difficile*, VAP, and CLABSI reduced significantly in 2019 after AMS program was implemented for almost 4 yrs in comparison to pre- AMS program (for *C. difficile*, reduced by 86.17%, *p* = 0.024; for VAP, reduced by 75%, *p* = 0.001, for CLABSI, reduced by 94.12%, *p* = 0.000; respectively, Fig. 1).

As the antibiotic consumption decreased after AMS implementation, the proportion of HAIs at the HMG facilities correspondingly decreased. The total consumption of antibacterial agents each year during the study period had a significant positive relationship with HAIs in hospitalized ill patients at the time of admission each year. The between-period difference in rates of health associated CLABSI infections was fluctuating possibly because of the non-adherence of prescribers to AMS guidelines, overuse, or improper use of antibiotics.

**Fig. 2:**
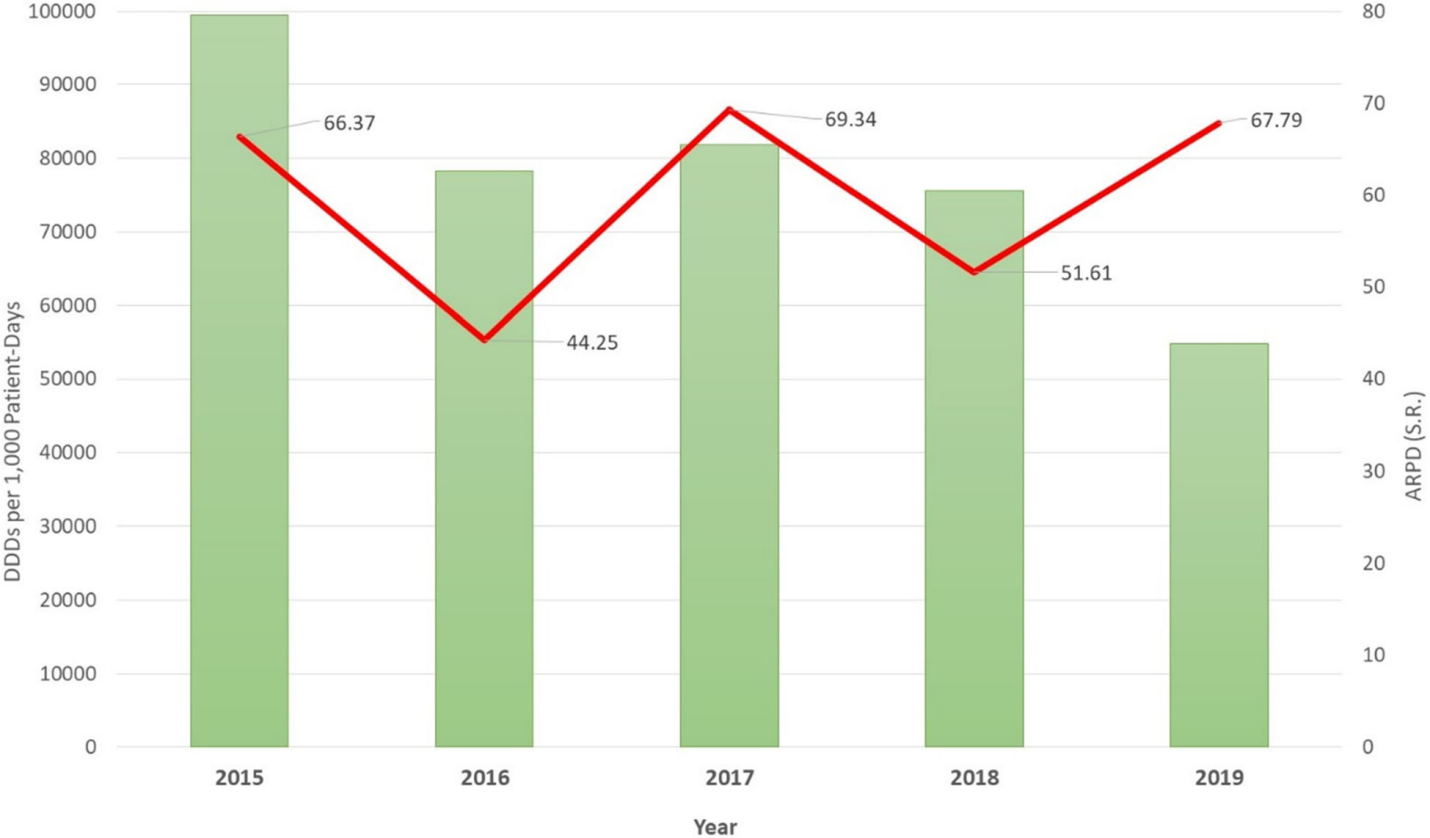
Total restricted antimicrobials consumption in all four HMG medical facilities before and after implementation of AMS Program, expressed as defined daily doses (DDDs) per 1000 patient-days (bars) and antimicrobial riyals per patient day (ARPD, indicated by solid line)

After the initiation of the AMS program in 2016, total DDDs use of all restricted antimicrobials values generally declined. Average total DDDs of all antimicrobials use for the period from 2016 to 2019 was lower than the total DDDs of all antimicrobials consumption in 2015 (72,690 vs 99,420 DDDs). Total DDDs consumption of the all antimicrobials in 2015 fell remarkably by 44.8% in 2019. Total antimicrobial consumption and antimicrobial riyals per patient-day before and after AMS initiation is shown in Fig. 2.

ARPD, which had risen steadily before AMS start, decreased by 33.32% in the first year after AMS implementation (2016), but increased by 4.48% in 2017; this trend was due almost exclusively to the continuous increased use of ciprofloxacin experienced over a period of several years. ARPD fell by 22.23% in 2018; and rose by 2.14% in 2019, a similar ARPD pre- AMS implementation, attributed to the biggest DDDs consumption for ciprofloxacin of all restricted antimicrobials throughout the whole 5 yrs (*p* = 0.648) (Table 1). Use of ciprofloxacin was high due to many advantageous pharmacokinetic properties including high oral bioavailability, large volume of distribution, and broad-spectrum antimicrobial activity against aerobic, enteric gram-negative bacilli (eg, Enterobacteriaceae, including *Escherichia coli*, Klebsiella spp., Proteus spp) [20]. Another reason for intravenous ciprofloxacin high usage was related to the practice of using the drug as a combination therapy with other anti-infectives in hospitalized patients (eg, diabetic foot, intra-abdominal, and pneumonia infections) [21].

**Table 1:** Estimated cost savings and expenses associated with the AMS program implemented at the four HMG medical facilities between 2016 and 2019

**year**	**Total antimicrobial expenditures (S.R.)**	**Patient-Days**	**ARPD (S.R.)**^**a**^	**Actual savings and expenses compared with 2015 (S.R.)**^**b**^
**2015**	11,502,287	173,297	66.37	
**2016**	8,229,378	185,958	44.25	4,113,261
**2017**	9,163,568	132,150	69.34	- 392,342
**2018**	7,601,056	147,269	51.61	2,173,668
**2019**	5,361,239	79,075	67.79	- 112,774

^a^ARPD = antimicrobial riyals per patient-day

^b^Calculated by subtracting the year’s ARPD from 2015’s ARPD and multiplying the result by the number of patient-days for the year. Total actual savings and expenses for 2016–2019, S.R. 6,286,929 (high savings) and S.R. 505,115 (low expenses)

For 2016–2019, the actual cost savings and expenses attributable to AMS activities ranged from S.R. 2,173,668 to S.R. 4,113,261 considered as high savings, and S.R. 392,342 to S.R. 112,774 supposedly regarded as low expenses.

## Discussion

Metrics for assessing the impact of AMS program on antibiotic use within a healthcare facility are essential part of any AMS strategy to identify problems or evaluate the benefits of AMS interventions [5, 6, 9–13]. Because assessing all indicators is unrealistic, we were encouraged to select the most relevant and feasible metrics for our particular settings. We utilized the DDDs, the most common and sustainable measure and least laborious way [22], which is suitable for the age category of our study participants [23].

A main finding in this study is the high consumption of ciprofloxacin that confirms the results of previous studies on using this restricted broad-spectrum antibiotic within Saudi Arabia [24–26]. Resistance to ciprofloxacin is growing locally [25, 27, 28] and worldwide [29, 30]and is commonly reported in most target bacteria [25, 27–30], use of ciprofloxacin should be generally reserved for complicated infections in which the benefits of use clearly outweigh the risks. The best approach to control the growing resistance is to control the use of ciprofloxacin and other fluoroquinolones use coupled with adherence to infection control measures to prevent spread of resistant strains among patients [5]. Surveillance of resistance pattern of prevalent strains and reduction of antibiotic consumption are essential for hospital prescribing policy and use of antibiotics [6].

Comparison of the antimicrobials consumption at the HMG health settings expressed as the DDDs per 1000 patient-days in this study with local data is not possible or very hard. Differences are not only limited to the total amount consumed but also related to the various antimicrobial classes used [9, 10, 24, 26, 31]. Comparing HMG findings on antimicrobials DDDs consumption to local data may help to identify hospital units or wards of highest use, to monitor the impact for future interventions, and to feedback prescribing physicians with the prescription patterns [26].

Currently, only one local study reported on the comparable efficacy of an AMS program on antimicrobial DDDs consumption per 1000 patient days before and after the intervention among hospitalized patients [9]. In general, findings of local studies on antimicrobial consumption are incomparable to our data because they differ in study design (retrospective [9, 24, 31] or prospective [10, 26]), type of AMS interventions (formulary restriction; preapproval strategies; and prospective audit and feedback [9], cascade reporting of antimicrobial susceptibilities (antibiogram); education; automatic stop orders; and formulary restriction [10], no specific AMS interventions were made [24], education; antibiogram; iv-to-oral switch; de-escalation; dose adjustment; and therapeutic drug monitoring of vancomycin [31], guidelines; formulary restriction; and activated AMS team [26]), age group (adults only [9, 26, 31], or children and adults [10, 24]), type of setting (medical 20-bed ICU (of 894-bed tertiary hospital) [9], general 5-bed ICU (of 380-bed general hospital) [10, 24, 31], tertiary 11-bed ICU (of 1000-bed hospital) [26]), antimicrobial consumption metric type (DDDs / 1000 patient-days [9, 10], DDDs / 100 patient-days [24, 31], DDDs and DOTs / 1000 patient-days [26]), and antimicrobial selection (≤ 5 restricted broad-spectrum agents [9], or > 5 restricted broad-spectrum agents [10, 24, 26, 31]). However, the average consumption of piperacillin/tazobactam, colistin, and tigecycline expressed in DDDs per 1000 patient-days post- AMS program in this study was a lot lower than the rates reported in a local report (21 vs 145, 10 vs 117, and 17 vs 21, respectively) [26].

In addition, we report much lower average consumption of ciprofloxacin and imipenem/cilastatin than rates reported in another local study when we convert their findings for the use of those two agents to be in DDDs per 1000 patient-days (72 vs 820, and 10 vs 52, respectively) [24]. Benchmarking antimicrobial consumption can improve the value of these data sources and can lead to more tailored and effective reduction measures.

Variations of antimicrobials consumption can be due to multiple factors, such as demographic characteristics, patient mix and severity of illness, awareness and education of healthcare providers and/or if they adhere to AMS guidelines. These differences should be taken into account when aiming to implement targeted interventions to reduce antimicrobial consumption.

The results of our study are in line with many studies that reported a statistically significant reduction in the rate of *C. difficile* [32–35], VAP [36–38], and CLABSI [39–41] following AMS implementation and showed that AMS interventions were associated with shorter duration of antibiotic therapy, less inappropriate antimicrobial use, and neutral effect in healthcare associated infections rates.

The implementation of the AMS program was associated with a decrease in the overall use of antimicrobials and a decrease in antimicrobial expenditures as reported in similar studies [42–44]. However, there are many indirect expenses which are expected to decrease proportionally, such as from antibiotic side effects and resistance, earlier transition to oral therapy, the discontinuation of unnecessary antimicrobial agents, increased length of hospital stay and readmission, and hospital-acquired infections which should be taken into consideration to support the implementation of AMS program.

## Limitations

The success of AMS program at HMG facilities must be considered in the context of several limitations. First, our study was limited by its pre-post quasi-experimental design, an empirical study used to estimate the causal impact of AMS program intervention on target population without random assignment. The findings could therefore suggest an association between AMS activities and reduced HAIs infection rates but could not establish definite causality. However, we believe the use of that design methodology was appropriate, as the random assignment of patients to an experimental group is generally not feasible.

Second, lack of demographic data of participants that would enhance applicability of study findings and attain a higher level for reporting key variables needed for conclusions and interpretation. Third, our study was not a true cost–benefit analysis but instead only provided an overview of the program’s implementation, resource utilization, and observed benefits.

Fourth, the AMS program was implemented without waiting to capture baseline quality data for longer than a year. Finally, secondary outcomes such as changes in length of stay of patients or readmission within 30 days after discharge were not calculated due to the difficulty in capturing the data for this subpopulation via electronic record query. Although antimicrobial resistance patterns were collected, evaluation of the AMS impact on resistance patterns was not included because that is beyond the scope of this paper. However, in almost all cases, trends were detected that suggested that the AMS program had a remarkable impact on decreasing antimicrobial utilization and cost while increasing quality of care.

## Conclusion

Antimicrobial stewardship program implementation at four Habib Medical Group (HMG) medical facilities was associated with a reduction in the consumption and cost of several broad-spectrum antimicrobial agents. It was also associated with lower incidence of healthcare associated infections within these hospitals, namely *Clostridium difficile* (*C. difficile*), ventilator-associated pneumonia (VAP), and central line-associated bloodstream infection (CLABSI). It might be the time to adopt such a program at a national level in all Saudi healthcare institutes to improve the quality of care provided and enhance patient safety measures. Future studies should analyze each component of AMS programs separately, while long-term evaluation of the effect of AMS programs is also warranted to determine their lasting influence on mortality and infection rates.

## Data Availability

Data are available upon request, please contact author for data requests.

## Abbreviations

AMS: Antimicrobial Stewardship
HAIs: Healthcare Associated Infections
VAP: Ventilator Associated Pneumonia
CLABSI: Central Line Associated Bloodstream Infection
DDD: Daily defined dose
HMG: Habib Medical Group
AMR: Antimicrobial Resistance
NAP: National Action Plans
UAE: United Arab Emirates
ARPD: Antimicrobial Riyals per Patient Day
ANOVA: Analysis of Variance

## References

[1] WHA68 R. 7. Global action plan on antimicrobial resistance. Sixty-eighth World Health Assembly, Geneva. 2015 May;26.

[2] A/71/L.2. Political Declaration of the high-level meeting of the General Assembly on antimicrobial resistance. New York: United Nations; 2016.

[3] Mendelson M, Balasegaram M, Jinks T, Pulcini C, Sharland M (2017). Antibiotic resistance has a language problem. Nature News.

[4] Dyar O, Huttner B, Schouten J, Pulcini C (2017). What is antimicrobial stewardship?. Clin Microbiol Infect.

[5] Davey P, Brown E, Charani E, Fenelon L, Gould IM, Holmes A, et al. Interventions to improve antibiotic prescribing practices for hospital inpatients. Cochrane Database Syst Rev. 2013;4. 10.1002/14651858.CD003543.pub3 23633313

[6] Schuts EC, Hulscher ME, Mouton JW, Verduin CM, Stuart JWC, Overdiek HW (2016). Current evidence on hospital antimicrobial stewardship objectives: a systematic review and meta-analysis. Lancet Infect Dis.

[7] Organization WH. Antimicrobial resistance: a manual for developing national action plans. 2016.

[8] Baur D, Gladstone BP, Burkert F, Carrara E, Foschi F, Döbele S (2017). Effect of antibiotic stewardship on the incidence of infection and colonisation with antibiotic-resistant bacteria and Clostridium difficile infection: a systematic review and meta-analysis. Lancet Infect Dis.

[9] Amer MR, Akhras NS, Mahmood WA, Al-Jazairi AS (2013). Antimicrobial stewardship program implementation in a medical intensive care unit at a tertiary care hospital in Saudi Arabia. Ann Saudi Med.

[10] Al-Tawfiq JA, Momattin H, Al-Habboubi F, Dancer SJ (2015). Restrictive reporting of selected antimicrobial susceptibilities influences clinical prescribing. J Infect Public Health.

[11] Alghamdi S, Shebl NA, Aslanpour Z, Shibl A, Berrou I (2018). Hospital adoption of antimicrobial stewardship programmes in gulf cooperation council countries: a review of existing evidence. J Glob Antimicrob Resist.

[12] Pate PG, Storey DF, Baum DL (2012). Implementation of an antimicrobial stewardship program at a 60-bed long-term acute care hospital. Infect Control Hosp Epidemiol.

[13] Borde J, Litterst S, Ruhnke M, Feik R, Hübner J, Dewith K (2015). Implementing an intensified antibiotic stewardship programme targeting cephalosporin and fluoroquinolone use in a 200-bed community hospital in Germany. Infection..

[14] Van Dijck C, Vlieghe E, Cox JA (2018). Antibiotic stewardship interventions in hospitals in low-and middle-income countries: a systematic review. Bull World Health Organ.

[15] Karanika S, Paudel S, Grigoras C, Kalbasi A, Mylonakis E (2016). Systematic review and meta-analysis of clinical and economic outcomes from the implementation of hospital-based antimicrobial stewardship programs. Antimicrob Agents Chemother.

[16] Lutters M, Harbarth S, Janssens JP, Freudiger H, Herrmann F, Michel JP (2004). Effect of a comprehensive, multidisciplinary, educational program on the use of antibiotics in a geriatric university hospital. J Am Geriatr Soc.

[17] Dellit TH, Owens RC, McGowan JE, Gerding DN, Weinstein RA, Burke JP (2007). Infectious Diseases Society of America and the Society for Healthcare Epidemiology of America guidelines for developing an institutional program to enhance antimicrobial stewardship. Clin Infect Dis.

[18] Drew RH, White R, MacDougall C, Hermsen ED, Owens RC (2009). Insights from the Society of Infectious Diseases Pharmacists on antimicrobial stewardship guidelines from the Infectious Diseases Society of America and the Society for Healthcare Epidemiology of America. Pharmacotherapy.

[19] World Health Organization. Anatomical Therapeutic Chemical/Defined Daily Dose Classification System. Available on: https://www.whocc.no/atc_ddd_index (Accessed on: 15 December 2019).

[20] Campoli-Richards DM, Monk JP, Price A, Benfield P, Todd PA, Ward A (1988). Ciprofloxacin Drugs.

[21] Ciprofloxacin (systemic): Drug information. Lexi-Drugs. Lexicomp. Wolters Kluwer Health, Inc. Riverwoods, IL. Available on: https://www.uptodate.com/contents/ciprofloxacin-systemic-drug-information?search=ciprofloxacin%20combination&topicRef=9267&source=see_link (Accessed on: 25 December y).

[22] Stanić Benić M, Milanič R, Monnier AA, Gyssens IC, Adriaenssens N, Versporten A (2018). Metrics for quantifying antibiotic use in the hospital setting: results from a systematic review and international multidisciplinary consensus procedure. J Antimicrob Chemother.

[23] World Health Organization. Definition and general considerations. Available on: https://www.whocc.no/ddd/definition_and_general_considera (Accessed on: 25 December 2019).

[24] Al-Tawfiq JA (2012). Changes in the pattern of hospital intravenous antimicrobial use in Saudi Arabia, 2006–2008. Ann Saudi Med.

[25] Babay HAH (2007). Antimicrobial resistance among clinical isolates of Pseudomonas aeruginosa from patients in a teaching hospital, Riyadh, Saudi Arabia, 2001-2005. Jpn J Infect Dis.

[26] Balkhy HH, El-Saed A, El-Metwally A, Arabi YM, Aljohany SM, Al Zaibag M (2018). Antimicrobial consumption in five adult intensive care units: a 33-month surveillance study. Antimicrob Resist Infect Control.

[27] Al-Tawfiq JA (2006). Increasing antibiotic resistance among isolates of Escherichia coli recovered from inpatients and outpatients in a Saudi Arabian hospital. Infect Control Hosp Epidemiol.

[28] Memish ZA, Shibl AM, Kambal AM, Ohaly YA, Ishaq A, Livermore DM (2012). Antimicrobial resistance among non-fermenting gram-negative bacteria in Saudi Arabia. J Antimicrob Chemother.

[29] Bidell MR, Palchak M, Mohr J, Lodise TP (2016). Fluoroquinolone and third-generation-cephalosporin resistance among hospitalized patients with urinary tract infections due to Escherichia coli: do rates vary by hospital characteristics and geographic region?. Antimicrob Agents Chemother.

[30] Riddle MS, Connor BA, Beeching NJ, DuPont HL, Hamer DH, Kozarsky P (2017). Guidelines for the prevention and treatment of travelers’ diarrhea: a graded expert panel report. J Travel Med.

[31] Momattin H, Al-Ali AY, Mohammed K, Al-Tawfiq JA (2018). Benchmarking of antibiotic usage: an adjustment to reflect antibiotic stewardship program outcome in a hospital in Saudi Arabia. J Infect Public Health.

[32] Talpaert MJ, Gopal Rao G, Cooper BS, Wade P (2011). Impact of guidelines and enhanced antibiotic stewardship on reducing broad-spectrum antibiotic usage and its effect on incidence of Clostridium difficile infection. J Antimicrob Chemother.

[33] Aldeyab MA, Kearney MP, Scott MG, Aldiab MA, Alahmadi YM, Darwish Elhajji FW (2012). An evaluation of the impact of antibiotic stewardship on reducing the use of high-risk antibiotics and its effect on the incidence of Clostridium difficile infection in hospital settings. J Antimicrob Chemother.

[34] Fowler S, Webber A, Cooper B, Phimister A, Price K, Carter Y (2007). Successful use of feedback to improve antibiotic prescribing and reduce Clostridium difficile infection: a controlled interrupted time series. J Antimicrob Chemother.

[35] Valiquette L, Cossette B, Garant M-P, Diab H, Pépin J (2007). Impact of a reduction in the use of high-risk antibiotics on the course of an epidemic of *Clostridium difficile*-associated disease caused by the hypervirulent NAP1/027 strain. Clin Infect Dis.

[36] Pugh R, Grant C, Cooke RP, Dempsey G. Short-course versus prolonged-course antibiotic therapy for hospital-acquired pneumonia in critically ill adults. Cochrane Database Syst Rev. 2011;10. 10.1002/14651858.CD007577.pub2 21975771

[37] Dimopoulos G, Poulakou G, Pneumatikos IA, Armaganidis A, Kollef MH, Matthaiou DK (2013). Short-vs long-duration antibiotic regimens for ventilator-associated pneumonia: a systematic review and meta-analysis. Chest..

[38] Chastre J, Wolff M, Fagon J-Y, Chevret S, Thomas F, Wermert D (2003). Comparison of 8 vs 15 days of antibiotic therapy for ventilator-associated pneumonia in adults: a randomized trial. JAMA..

[39] Pronovost PJ, Watson SR, Goeschel CA, Hyzy RC, Berenholtz SM (2016). Sustaining reductions in central line–associated bloodstream infections in Michigan intensive care units: a 10-year analysis. Am J Med Qual.

[40] Jain M, Miller L, Belt D, King D, Berwick D (2006). Decline in ICU adverse events, nosocomial infections and cost through a quality improvement initiative focusing on teamwork and culture change. BMJ Quality & Safety.

[41] Murni IK, Duke T, Kinney S, Daley AJ, Soenarto Y (2015). Reducing hospital-acquired infections and improving the rational use of antibiotics in a developing country: an effectiveness study. Arch Dis Child.

[42] Bartlett JM, Siola PL (2014). Implementation and first-year results of an antimicrobial stewardship program at a community hospital. Am J Health Syst Pharm.

[43] Cook PP, Gooch M (2015). Long-term effects of an antimicrobial stewardship programme at a tertiary-care teaching hospital. Int J Antimicrob Agents.

[44] Taggart LR, Leung E, Muller MP, Matukas LM, Daneman N (2015). Differential outcome of an antimicrobial stewardship audit and feedback program in two intensive care units: a controlled interrupted time series study. BMC Infect Dis.

